# The Dockstore: enhancing a community platform for sharing reproducible and accessible computational protocols

**DOI:** 10.1093/nar/gkab346

**Published:** 2021-05-12

**Authors:** Denis Yuen, Louise Cabansay, Andrew Duncan, Gary Luu, Gregory Hogue, Charles Overbeck, Natalie Perez, Walt Shands, David Steinberg, Chaz Reid, Nneka Olunwa, Richard Hansen, Elizabeth Sheets, Ash O’Farrell, Kim Cullion, Brian D O’Connor, Benedict Paten, Lincoln Stein

**Affiliations:** Adaptive Oncology, Ontario Institute for Cancer Research, Toronto, Ontario M5V 3S1, Canada; UC Santa Cruz Genomics Institute, University of California Santa Cruz, Santa Cruz, CA 95060, USA; Adaptive Oncology, Ontario Institute for Cancer Research, Toronto, Ontario M5V 3S1, Canada; Adaptive Oncology, Ontario Institute for Cancer Research, Toronto, Ontario M5V 3S1, Canada; Adaptive Oncology, Ontario Institute for Cancer Research, Toronto, Ontario M5V 3S1, Canada; UC Santa Cruz Genomics Institute, University of California Santa Cruz, Santa Cruz, CA 95060, USA; UC Santa Cruz Genomics Institute, University of California Santa Cruz, Santa Cruz, CA 95060, USA; UC Santa Cruz Genomics Institute, University of California Santa Cruz, Santa Cruz, CA 95060, USA; UC Santa Cruz Genomics Institute, University of California Santa Cruz, Santa Cruz, CA 95060, USA; UC Santa Cruz Genomics Institute, University of California Santa Cruz, Santa Cruz, CA 95060, USA; UC Santa Cruz Genomics Institute, University of California Santa Cruz, Santa Cruz, CA 95060, USA; UC Santa Cruz Genomics Institute, University of California Santa Cruz, Santa Cruz, CA 95060, USA; UC Santa Cruz Genomics Institute, University of California Santa Cruz, Santa Cruz, CA 95060, USA; UC Santa Cruz Genomics Institute, University of California Santa Cruz, Santa Cruz, CA 95060, USA; Adaptive Oncology, Ontario Institute for Cancer Research, Toronto, Ontario M5V 3S1, Canada; Data Sciences Platform, Broad Institute, Boston, MA 02142, USA; UC Santa Cruz Genomics Institute, University of California Santa Cruz, Santa Cruz, CA 95060, USA; Adaptive Oncology, Ontario Institute for Cancer Research, Toronto, Ontario M5V 3S1, Canada

## Abstract

Dockstore (https://dockstore.org/) is an open source platform for publishing, sharing, and finding bioinformatics tools and workflows. The platform has facilitated large-scale biomedical research collaborations by using cloud technologies to increase the Findability, Accessibility, Interoperability and Reusability (FAIR) of computational resources, thereby promoting the reproducibility of complex bioinformatics analyses. Dockstore supports a variety of source repositories, analysis frameworks, and language technologies to provide a seamless publishing platform for authors to create a centralized catalogue of scientific software. The ready-to-use packaging of hundreds of tools and workflows, combined with the implementation of interoperability standards, enables users to launch analyses across multiple environments. Dockstore is widely used, more than twenty-five high-profile organizations share analysis collections through the platform in a variety of workflow languages, including the Broad Institute's GATK best practice and COVID-19 workflows (WDL), nf-core workflows (Nextflow), the Intergalactic Workflow Commission tools (Galaxy), and workflows from Seven Bridges (CWL) to highlight just a few. Here we describe the improvements made over the last four years, including the expansion of system integrations supporting authors, the addition of collaboration features and analysis platform integrations supporting users, and other enhancements that improve the overall scientific reproducibility of Dockstore content.

## Graphical Abstract

**Graphical Abstract:**
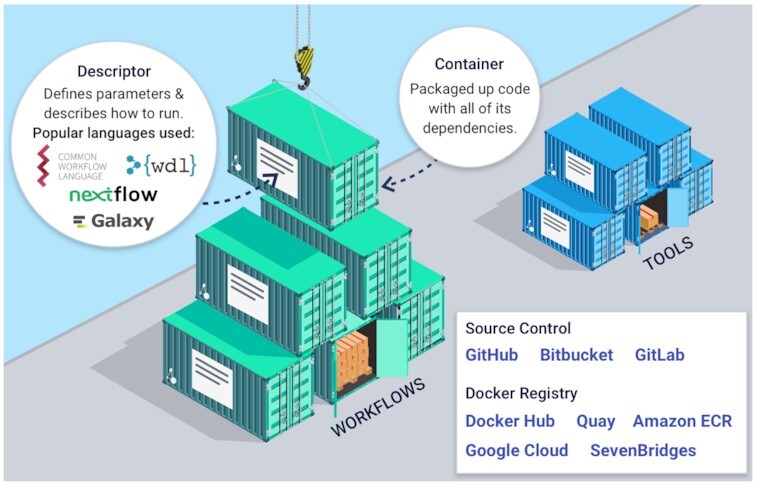
Dockstore: enhancing an open platform for sharing Docker-based tools described with workflow languages for the sciences.

## INTRODUCTION

Open Science in bioinformatics has enabled researchers to share and extend a wealth of computational methods. However, tapping into this shared knowledge is plagued by reproducibility issues that hinder the validation (1) of published results and stall overall scientific progress. Rather than building on resources created by domain experts, significant time is instead spent on overlapping efforts such as finding source code, setting up software and troubleshooting environment-specific dependency conflicts.

Solving these issues with software reuse and portability becomes increasingly significant as biomedical research shifts towards analyzing petabyte-scale data that is federated across institutions. For these datasets, cost concerns make data transfer infeasible, and in the case of personal genetic or health information, data transfer may be legally restricted. These considerations require collaborators to instead focus on moving algorithms and analysis across computing environments. Coordinating research at this scale involves heavy infrastructure management and overhead, often limiting participation to well-resourced groups with dedicated technical personnel.

We originally created Dockstore in response to similar challenges faced by the Pan-Cancer Analysis of Whole Genomes (PCAWG) study (2), which ran between 2014 and 2019. This project called for a common set of cancer variant-calling workflows to be run in a consistent and reproducible fashion across 14 different cloud and conventional computing infrastructures on an internationally federated set of whole cancer genomes totalling roughly 1 PB in size. Our innovative solution was to combine Docker, then a relatively new lightweight virtualization technology, with workflow languages that programmatically describe the step by step execution of the containerized software in a human readable way. Thus was born Dockstore, a standardized way of packaging, registering, finding and executing analysis workflows that provides improved reproducibility across multiple computing environments. Dockstore is comparable to single language workflow registries such as Agora and the Galaxy Toolshed (3) which are associated with a specific workflow platform, or with container registries such as BioContainers (4). Dockstore's main distinguishing characteristic is that it is a general solution not tied to any particular workflow architecture, language, or platform.

In this way, Dockstore aims to serve as a centralized library of computational methods for the growing variety of technologies that use workflow, container, and cloud solutions for the reproducibility, scalability, and portability of computational analysis in bioinformatics (5,6). Dockstore integrates with multiple workflow languages and ‘*Launch with*’ partners that can import and run workflows from Dockstore as a service, simplifying analysis and reducing technical barriers for end users.To date, >250 (7) workflow engines have been tracked and many require difficult configuration to run outside their home institutions. The workflow languages that we chose to support in Dockstore were selected due to the robust communities of these languages and like-minded ideas about reproducible workflows, provenance, and best practices in developing bioinformatics software.

To share a computational method on Dockstore, a developer first encapsulates the steps of a workflow's environment in a container (8) and then programmatically outlines the analysis steps using a workflow language (also referred to as a ‘descriptor’). Together these are registered into Dockstore via popular source control sites. A bioinformatician who wants to either validate this analysis or apply the methods to their own research simply has to search for the workflow and ‘*Launch with*’ into a platform with their own data.

The initial version of Dockstore is described in our 2017 publication (9). Over the past four years, Dockstore has continued building features that increase the Findability, Accessibility, Interoperability, and Reusability (FAIR) (10) of computational analysis resources with our ambition being to make the creation, sharing, and reproduction of scientific analyses as easy as the sharing of scientific publications. In the next sections we provide a brief summary of the main components of Dockstore and then continue with a detailed overview of our new and enhanced features.

### Dockstore core functionality

Dockstore aims at being a home for reproducible workflows, exchanged using standardized Global Alliance for Genomics and Health (11) (GA4GH) APIs, and developed in a well-integrated way with common software engineering principles and practices while facilitating their use by users across cloud execution platforms. We have designed Dockstore with two main user types: (i) The developer or author user is typically a bioinformatician or software engineer that will package up their methods using containers and descriptor languages to share tools and workflows with the scientific community. (ii) An end user is a researcher interested in using these ready-to-use resources for their own research applications or for reproducing and validating published results. With this in mind, the core of Dockstore includes:

#### Dockstore main site

Dockstore's keystone, and the focus of this paper, is the Dockstore website at https://dockstore.org/. It is driven by several components, including the back-end web service, the database, and a front-end user interface. These components work together to provide end users with the ability to register workflows, search for workflows, review the versions of workflows including information on cloud platforms that are able to launch them, and to stay abreast of relevant updates for followed users, organizations, and workflows.

#### Dockstore library

**Figure 1.**
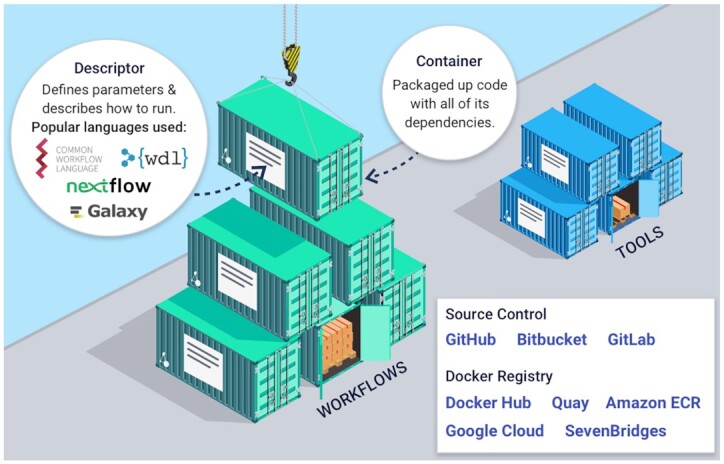
Dockstore makes computational analysis accessible and reproducible by combining containers, descriptor languages, and test parameter files to simplify software reuse and dependency management.

A workflow or tool in Dockstore is composed of three essential elements as in Figure 1, including (i) links to the underlying open-source code repository, such as Github or Bitbucket, (ii) links to the containers for the tools in a container repository, such as Docker Hub or Quay.io and critically (ii) the metadata and workflow language descriptions that describe how the tool is configured and parameterized to operate as a complete scientific analysis. By following these best practices, an entry on Dockstore can more easily be incorporated into larger workflows or even decomposed into smaller parts for custom use cases. Once a workflow or tool is registered with Dockstore, it is published in a searchable index where it can be found by other researchers.

#### Dockstore ecosystem

The Dockstore ecosystem consists of three separate important groups: (i) the workflow contributors, who provide valuable tools and workflows to Dockstore; (ii) the *Launch with* partners, who can run workflows with a few clicks and (ii) our development teams based at OICR and UCSC, which maintain the site and work on new features.

Dockstore provides numerous tutorials, tools, and gentle nudges that help workflow authors create reproducible workflows.We also provide many resources to help orient end users to the Dockstore ecosystem and further training for newcomers to container, cloud, and workflow technologies. A collection of these tutorials and their links can be found in the Supplemental Materials with topics such as:

- How to launch tools and workflows to Launch with platforms or through the Dockstore CLI
- Getting started with Docker and workflow languages
- Best practices for creating secure and FAIR tools, workflows, and containers
- Registration of Dockerfiles to allow for independent validation of the software environment
- Using checker workflows to automatically test that a workflow functions across multiple software environments in an automated fashion
- The ability to freeze a workflow into an immutable version with checksums tracked for reproducible use and the issuance of DOIs for citations

## NEW AND ENHANCED FEATURES

Dockstore development over the past four years has focused on expanding support for workflow languages and execution platforms, adding integrations with several open source software development environments (making it easier to write reproducible, well tested workflows), and enabling the creation of immutable workflows for publication referencing. In the sections below, we highlight several major improvements to the platform, while also briefly touching on a few smaller themes that impact much of our work A high level summary of how FAIR principles have been implemented in these improvements is shown in Table 1.

**Table 1.** Dockstore's support for FAIR principles

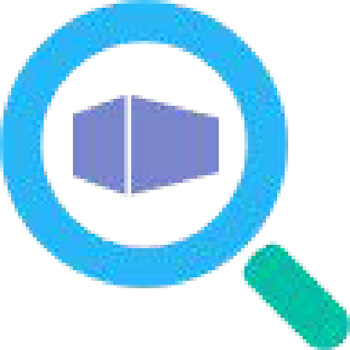	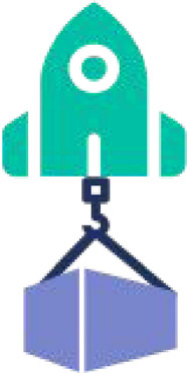	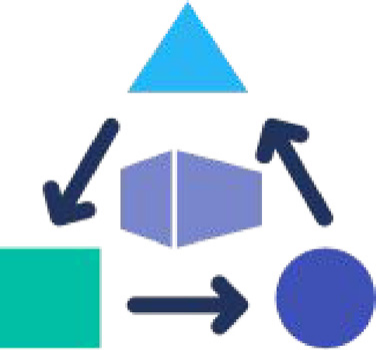	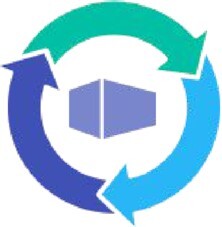
Findable	Accessible	Interoperable	Reusable
All runtime needs and metadata are packaged together, parsed, and indexed for robust searching with the option to generate DOIs.	Dockstore never requires a user to login to search and inspect contents for workflows and tools. Links to source repositories always provided.	Standardized APIs and agnostic support of multiple languages and repositories enables the simple launching of workflows to a variety of compute platforms.	Ready-to-use, version controlled portability using containers and human readable workflow languages with provided test files and documentation to simplify reproducibility.

### ‘*Launch with*’ partners

One of the most distinguishing features introduced to Dockstore has been its integration with a variety of cloud platforms to directly launch and run workflows over a browser interface. These platforms cover a selection of academic and commercial institutions, each of which offers a variety of datasets and features. In most cases, integration works by allowing users to click-through from the Dockstore site to the *Launch with* partner site. The partner site then uses the GA4GH TRS (12) API to retrieve information on the selected workflow version, collects additional runtime parameter information from the user, and then executes the workflow using its Docker image and workflow description. Some partners have also implemented support for directly browsing TRS-enabled services from within the partner site. Information passed over TRS includes workflow descriptors and can also include examples of parameter files or checksums to verify that Docker images have not been tampered with.

Cloud platforms shift infrastructure management to service providers, enabling bioinformaticians to focus on the core of their research, rapidly scale analysis as needed, while also reducing the overhead requirements for dedicated technical personnel. Several of these platforms also address the privacy and legal concerns regarding the access and transfer of patient data, such as meeting the regulatory standards for FedRAMP (13) authorization and HIPPA compliance, and also integrating with verification services to facilitate a researcher's existing access to controlled datasets. Table 2 summarizes the features offered by our *Launch with* partners to date. It should be noted that as these platforms evolve to support newer versions of CWL (14) and WDL (15), Dockstore has updated its validation and testing to match with support for CWL 1.1 and WDL 1.0 as of publication.

**Table 2.** For the WDL (10) workflow language, Dockstore offers *Launch with* DNAstack (https://www.dnastack.com/), DNAnexus (https://www.dnanexus.com/), Terra (https://terra.bio/), FireCloud (http://firecloud.terra.bio) through Terra's integration, NHLBI Biodata Catalyst (https://biodatacatalyst.nhlbi.nih.gov/), and AnVIL (https://anvilproject.org/). For the CWL (11) workflow language, Dockstore offers *Launch with* the Cancer Genomics Cloud (https://www.cancergenomicscloud.org/), Cavatica(https://cavatica.squarespace.com/) powered by Seven Bridges Genomics, and NHLBI Biodata Catalyst

Cloud platform	Languages	Academic or commercial	Browser launch from Dockstore	Launch from within Cloud Platform
DNAstack	WDL	Commercial	yes	Yes
DNAnexus	WDL	Commercial	yes	
Terra	WDL	Academic	yes	
Firecloud	WDL	Academic	yes, via Terra ‘*Launch with*’	
Cancer Genomics Cloud (CGC)	CWL	Partnership	yes	
AnVIL	WDL	Academic	yes	
NHLBI BioData Catalyst	CWL, WDL	Partnership	yes	
Cavatica	CWL	Partnership	yes	
Galaxy Project	Galaxy	Academic		Yes

### Nextflow and galaxy support

Recently, Dockstore has added support for the Nextflow (16) and Galaxy (17) workflow languages. Support for a workflow language on Dockstore means that at minimum, we can do some light parsing of workflow content as a sanity check while registering workflows in that language. The team is currently working toward the level of support that we have for WDL and CWL (generating and displaying Docker images used by a workflow, generate directed acyclic graphs to display workflow structure, and display one-click ‘*Launch with*’ buttons to allow the user to quickly run a workflow on compatible workflow platforms). Details on our level of support for a workflow language is kept up to date on our documentation site (https://docs.dockstore.org/en/develop/end-user-topics/language-support.html).

In addition to supporting the Galaxy workflow language, Dockstore has also started using a plugin architecture that has allowed the project to solicit and quickly integrate code contributions from the Galaxy team. In the future, broader use of this interpreter design pattern (18) via plugins will allow for a more streamlined and rapid expansion of workflow language support on Dockstore.

### Source control integrations and GitHub apps, search

**Figure 2.**
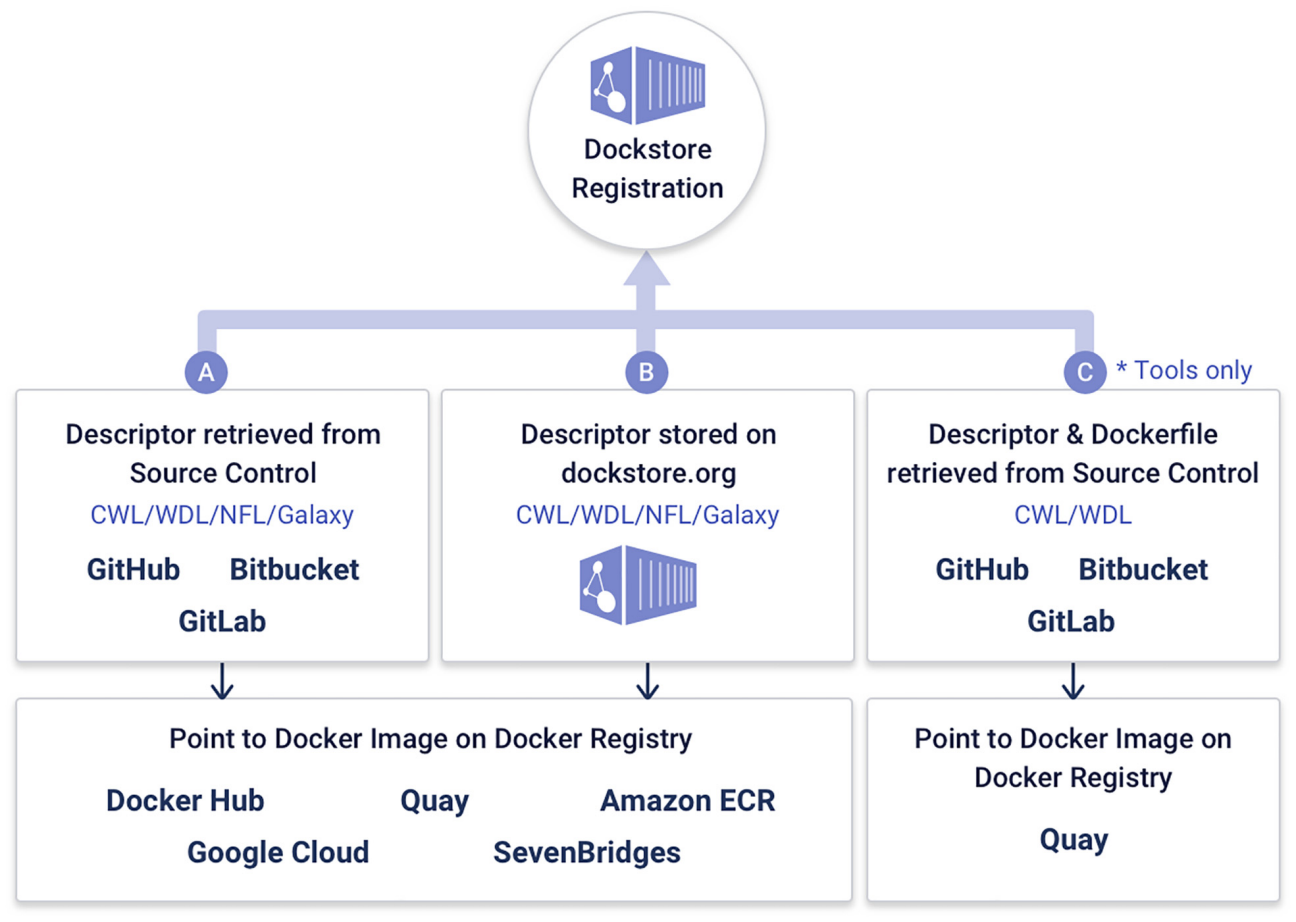
Dockstore can register workflows in three main ways, from source control, stored on dockstore.org directly, or tools with descriptors found via quay.io.

One of the features that distinguishes Dockstore from other platforms is the ability to synchronize workflows from source control repositories like GitHub, BitBucket, and Gitlab. This allows developers to maintain their current development practices while also placing their workflows into a centralized and findable repository that enhances sharing and re-use within the bioinformatics community. This integration is illustrated in Figure 2.

A recent addition to the platform is the ability to automatically synchronise new versions of workflows from GitHub without requiring developers to visit the Dockstore site. In the traditional method of registering workflows on Dockstore, authors sync their workflows from source control initially, but have to return to Dockstore to pull new versions or releases of their workflows. By using the Dockstore GitHub app (https://docs.dockstore.org/en/develop/getting-started/github-apps/github-apps.html), users are able to produce new releases of their software and have these updates automagically pushed to Dockstore.

### Snapshots, checksums, DOIs

One challenge for reproducible analysis is the difficulty in assessing whether a workflow that one user ran is precisely the same as a workflow run by another user. Dockstore's initial approach to this challenge was incomplete since it relied solely on source control version numbers, which allowed users to delete workflows with one version number and then reuse the same version number for a different workflow. Similarly, the original version of Dockstore allowed users to delete the Docker images that a particular workflow relied on and re-upload something different.

Dockstore now collects information that detects and prevents sources of inconsistency while also enhancing security. Workflow authors can trigger a feature known as a snapshot that makes a version of a workflow collected by Dockstore immutable, meaning it is no longer subject to change when synchronising with source control. We also collect checksum information for both the workflow language descriptors and for the Docker images used, which can detect when users attempt to run workflows that have been altered after the snapshot event. This functionality was introduced and exposed in Dockstore's implementation of version 2.0 of the GA4GH TRS standard.

While implementing this feature, Dockstore added an integration that allows workflow authors to upload their immutable snapshot to Zenodo (https://about.zenodo.org/) which generates a digital object identifier (DOI), allowing users to cite workflows in publications (19). This encourages workflow developers and interested parties to think of their source code as real and legitimate products of their research, while also making it easier to cite them along with any academic work.

### GA4GH linkages and checker workflows

**Figure 3.**
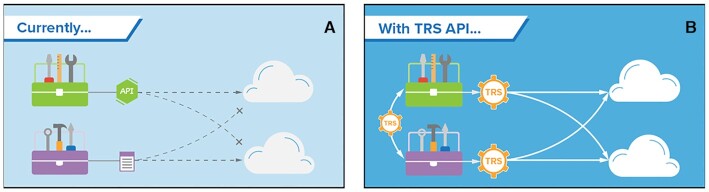
Dockstore contributed to the development of GA4GH TRS, an API that it uses for distributing workflows to *Launch with* partners and tools such as CWL’s cwltool. (**A**) Currently, cloud analysis environments use proprietary APIs or custom scripts to access tools. This makes it difficult to publish tools in one place and use them in different cloud analysis environments. (**B**) The TRS (Tool Registry Service) API provides a standard way to retrieve standard workflows from multiple cloud environments. TRS also provides a channel for different groups to share tools. Courtesy of Stephanie Li, GA4GH.

The Dockstore team contributes to a number of GA4GH standards and is the leading implementation for the Tool Registry Service (TRS) standard to aid in interoperability with other software projects in the genomics and health community. As a part of the Cloud Work Stream, Dockstore's implementation of TRS allows the platform to provide listings and search for tool and workflow information to its *Launch with* partners and, potentially, collaborating workflow registries (Figure 3). This standard became an official GA4GH standard in October 2019 and Dockstore implements two draft standards and the official 2.0.0 version of TRS.

The Dockstore team also helped organize the GA4GH-DREAM Workflow Execution (https://doi.org/10.6084/m9.figshare.6716063.v1) challenge in 2017. As a part of this challenge participants contributed Dockstore-style workflows and manually ran them across a variety of environments. Dockstore integrated the ability to register and search for checker workflows (https://docs.dockstore.org/en/develop/advanced-topics/checker-workflows.html), workflows that test themselves across a variety of platforms. Like regular workflows, checker workflows are workflows, but they examine the output of a ‘real’ biologically significant workflow to determine whether it ran correctly in a new computing environment, explicitly testing for scientific reproducibility.

### Support for the social aspects of community software development

Dockstore owes much to its user community. One of the earliest and most prominent feature requests was to give groups a way to showcase their tools and workflows while also giving users a better sense of trust by knowing who uses or ‘vouches’ for the quality of particular resources.

Organizations and Collections are new features that allow institutions, companies, grant funding agencies, and collaborations to highlight the tools and workflows that they either create themselves or find on Dockstore for re-use. This is somewhat analogous to a playlist on a music/video streaming platform that allows prominent users to curate content that they find enjoyable.

To create an Organization on Dockstore, a representative submits a request on behalf of the group for the Dockstore team to verify and review. In addition to creating a place for these organizations to highlight their work, this also provides a place to provide both free-form Markdown descriptions of their work and to impart some trust by tying together user identities (solidified by their GitHub user profiles or by an ORCID (20) profile) with the tools and workflows that they have curated.

Another social process that the Dockstore team manages in order to provide more trust in the quality of workflows is verification. Workflows are considered verified when the Dockstore team has contacted a third party (independent of the original authors) that can confirm that a workflow runs as intended with a provided parameter file and public input data.

Finally, a dashboard provides returning users with both curated and customised content that may be of interest. Curators create notifications and listings of featured content that may be topical. For example, at the time this was written, a collection of COVID-19 workflows (https://dockstore.org/organizations/BroadInstitute/collections/pgs) was highlighted in this fashion. The dashboard also included automatically customized content such as updates on previously starred (bookmarked) workflows that have received updates and shortcuts to the returning user's own content.

### Core infrastructure and site reliability

Building confidence in the tools and workflows is always a challenge for a public repository. As a result, we have developed processes to strengthen site availability and to help ensure that content cannot be modified by unauthorised users. We have adopted infrastructure as code (21) practices such as capturing the platform's configuration using templates for simplified deployment and site management. We have also upgraded to newer versions of Angular and Java (22) while also implementing a robust suite of automated testing frameworks for continuous integration across all parts of Dockstore's development stack. During 2020 and 2021 we also have been working on improving our deployment and security processes in an effort toward achieving FedRAMP compliance. This will include increasing the robustness and availability of our infrastructure as a whole while allowing Dockstore to interoperate with a wide variety of secure computing environments within North America that handle personal health information (PHI).

## COLLABORATIONS AND COMMUNITY ORGANIZATIONS

At the time of publication there have been 705 workflows and 240 tools published to the Dockstore library along with >20 Organizations. As a result, we wish to highlight a few partner collaborations and community groups that have contributed workflows or integrations with Dockstore.

### NHLBI BioData catalyst

NHLBI BioData Catalyst (https://biodatacatalyst.nhlbi.nih.gov) is a cloud-based ecosystem that provides workflows and data for analysis in secure workspaces to enable and accelerate research using rich data resources related to heart, lung, blood and sleep diseases. As one of our *Launch with* partners, the NHLBI BioData Catalyst project uses Dockstore as its official repository for their created and preferred workflows. Users can launch any valid CWL or WDL workflow, including those maintained directly by the BioData Catalyst community found on their Organization page at https://dockstore.org/organizations/bdcatalyst.

One of these showcased collections include the workflows and tools used for generating and analyzing the 53,831 diverse genomes from the NHLBI Trans-Omics for Precision Medicine(TOPMed) Program (23). The TOPMed Aligner and Freeze 3 and 5b/8 Variant Caller workflows allow users to exactly reproduce the analysis done on samples from the TOPmed dataset or user's can bring their own data to align and variant call new short-read sequencing samples based on the methods used in the study. In collaboration with BioData Catalyst for outreach and training on our integrated ecosystems, we’ve created a tutorial to show users how to launch these workflows with real data in a cloud environment (https://app.terra.bio/#workspaces/biodata-catalyst/TOPMed%20Aligner%20Gen3%20Data).

We further worked with early BioData Catalyst users to create and highlight reproducible genomic analysis downstream of variant calling using community resources. These collections include Genome-Wide Association Studies (GWAS), Structural Variant Calling workflows, and others.

### NHGRI AnVIL

Dockstore is one of the platform components for the National Human Genome Research Institute's (NHGRI) Genomic Data Science Analysis, Visualization, and Informatics Lab-Space (AnVIL), a cloud resource also working on bringing users to a unified cloud computing environment with rich datasets, workflows, and scalable, shared computing resources (anvilproject.org). Dockstore serves as the official repository for the tools and workflows that can be directly launched into AnVIL’s Terra powered compute environment which also nests a Galaxy platform.

Since 2019, Dockstore and AnVIL has also been working to support the sharing and launching of Galaxy workflows into AnVIL. AnVIL is compiling relevant workflows for their community using the Dockstore organization feature (https://dockstore.org/organizations/anvil). Workflows shared include the GWAS best practice pipeline used in the consortium.

### Highlighted community organizations and workflow collections

Dockstore's new Organizations and Collections features have enabled groups to showcase both their own workflows and the workflows that they use from others. We would like to highlight a few that demonstrate a high degree of utility for researchers, are of topical interest, or demonstrate a special degree of integration with Dockstore. These workflows and organizations are created by the community and are not the result of direct partnerships with Dockstore staff.

#### Viral Genomics (COVID-19) & GATK best practice workflows

The Broad Institute's Viral Genomics (24) collection provides ready-to-use workflows for the assembly, QC, metagenomics, and data analysis of viral genomes. These workflows are automatically updated from their underlying source repositories using the Dockstore GitHub app. The workflows let users work with either their own data or with public data pulled directly from NCBI SRA and Genbank. To also facilitate sharing and simplify open data collaboration, a provided utility workflow automates the preparation and bulk upload of data files to GenBank. The workflows, documentation, and tutorials for their use in COVID-19 genomic analysis are provided and maintained by the Broad Viral Genomics & Data Sciences Platform and can be found on Dockstore on the Broad Institute organization page.

Other prominent collections shared by the Broad Institute on Dockstore include the GATK best practices (25) workflows (https://dockstore.org/organizations/BroadInstitute/collections/GATKWorkflows). These workflows are widely used in the genomics community and can be launched on WDL *Launch with* partners and a tutorial is available for the Broad's Terra platform (26).

#### nf-core

Uniformly registered onto Dockstore using GitHub apps, the nf-core workflows (27) are another good example of a set of workflows that are automatically updated on Dockstore.The nf-core organization (https://dockstore.org/organizations/nfcore) of workflows represents a community effort to build a curated set of high quality workflows in the Nextflow language. The nf-core workflows are a particularly good fit for the goals of Dockstore since all are validated using continuous integration to ensure reproducibility, are uniformly documented from a shared template, and encourage benchmarks on cloud environments. Workflows are available for a wide variety of use cases such as RNA-seq analysis, proteomics, nanopore sequencing, and more.

#### PCAWG and GA4GH-DREAM workflows

Other prominent collections include the workflows from the Pan-cancer Analysis of Whole Genomes (PCAWG) and the GA4GH-DREAM workflow execution challenge (https://www.synapse.org/!Synapse:syn8507133/wiki/415976). The latter challenge was a particularly good fit since the goal of the exercise was to test that Dockstore-style workflows would function across multiple cloud platforms. Subsequently, the Dockstore team has included 24 of these workflows in an automated testbed for evaluating new versions of the Dockstore platform. This helps to ensure backwards compatibility and the output logs are also available to end-users to help with debugging.

## USER SUPPORT, TRAINING, AND OUTREACH

As the Dockstore site has grown in popularity, the development team has also increased the ways in which we provide support for workflow developers, users of workflows and integration contributors. The team has created a large corpus of publicly available tutorials, FAQs, and training videos, available at https://docs.dockstore.org/en/develop/posters-and-talks.html. This includes documentation covering all the core features of the site, as well as developer documentation and user-focused tutorials for authoring workflows with Dockstore.

Users can also post their questions to our built-in discussion board (https://discuss.dockstore.org/) or via issues opened on our open-source code repository on GitHub (https://github.com/dockstore/dockstore). The public forum provided by these venues allows both the Dockstore team and the community at large to collaborate towards and benefit from shared solutions.

An important part of Dockstore is not just to build the site, but to provide live tutorials about best practices while also keeping in touch with the community to understand how the field is developing. The Dockstore team has given presentations at a large number of North American and International conferences such as our BOSC talks in 2017 and 2019. Additionally, we have conducted a number of online training sessions as part of Canadian Bioinformatics Workshops (CBW), with Terra, at BOSC 2020, and with many of our *Launch with* partners.

In these trainings, we aim to provide users with a basic understanding of Docker, cloud technologies, and sharing workflows on Dockstore. Traditional learning resources for container and workflow technologies are typically designed for those with conventional software engineering backgrounds and for use cases beyond the scope of analytical workflows. In our hands-on workshops we address the learning barrier this creates by focusing on building skills and best practices relevant to scientists and bioinformaticians, with the goal being to empower these users with the fundamentals knowledge to not only use, but to also create their own reproducible tools and workflows (See highlighted tutorials in supplementary material). These trainings have the added benefit of allowing the team to connect and collaborate with many like minded communities including Nextflow and Biocontainers (Elixir Europe).

The Dockstore team has also conducted training sessions for supported research groups that have added workflows to Dockstore. This includes groups sharing their tools and workflows with the community through Dockstore organization pages, such as the Large-Scale Gene by Environment analyses (https://dockstore.org/organizations/LSGxE) and the VG team at UC Santa Cruz who are pioneering tools for analyzing graph genomes (https://dockstore.org/organizations/UCSCGI/collections/variationgraphs).

### Future work

We see a number of promising avenues for Dockstore to grow and evolve in the future while continuing to break down silos between communities. Many of the new developments discussed here have not only enhanced the current Dockstore experience, but also lay the foundation to more robustly support and rapidly integrate new collaboration features, workflow languages and partner platforms.

As part of this future work, we hope to work with our partner cloud platforms and workflow contributors to share benchmarked runtime data to give users a more transparent guide to workflow usage and costs. Dockstore is also using the evolving GA4GH Workflow Execution Service (WES) standard to provide command-line users with a simplified way of launching workflows remotely onto novel cloud platforms via the Dockstore CLI (https://doi.org/10.5281/zenodo.4536482).

Dockstore also plans to expand its support of reproducible, portable software into new areas to give users the ability to package and launch full fledged applications and services. This would allow for the deployment and sharing of research infrastructure such as reference data servers, Jupyter notebooks, as well as visualization tools such as genome browsers.This functionality will complement tools and workflows by providing a means of sharing complete, web-based applications that enable interactive analysis by users. These features are already in early development, available to users as a public preview. The team is also looking into ways of improving the longevity of the Docker images used by workflows themselves, possibly by extending the snapshot and DOI feature.

Importantly, Dockstore is prioritizing efforts to harden the security and trust of our platform and the content shared within it. Completing regulatory compliance and improving our security is a top priority to better integrate with collaborators hosting controlled datasets. We also anticipate giving users an easier way to measure the quality of workflows in terms of adherence to best practices. We are also working with the NHLBI (National Heart, Lung, and Blood Institute) in the US to provide measures of quality (bronze, silver, and gold) for specific workflows dependent on whether they provide open access data, signed Docker containers, workflow code signing, and endorsements by known entities. This, in turn, will provide information to users and platforms running the workflows that will enable choices to be made at the level of trust ascribed to content from Dockstore.

## DATA AVAILABILITY

Dockstore is an open source collaborative initiative available in the GitHub repository (https://github.com/dockstore/dockstore) with supporting code nested under that GitHub organization. The production site is hosted at https://dockstore.org/ All published workflows and tools can be exported using the GA4GH TRS API.

## Supplementary Material

gkab346_Supplemental_File
