# Supplementary material for: The Dockstore: enhancing a community platform for sharing reproducible and accessible computational protocols
Source: Nucleic Acids Res. 2021 May 12;49(W1):W624–32. doi: 10.1093/nar/gkab346 (PMC8218198; doi:10.1093/nar/gkab346)
Supplement: gkab346_Supplemental_File [file gkab346_supplemental_file.pdf]

Supplemental Data

Highlighted documentation, tutorials, and trainings:

Launching Tools and Workflows	https://docs.dockstore.org/en/develop/launch-with/launch.html
Getting Started Guide	https://docs.dockstore.org/en/develop/getting-started/getting-started.html
Registering Your Workflow to Dockstore	https://docs.dockstore.org/en/develop/getting-started/dockstore-workflows.html#register-your-workflow-in-dockstore
Docker Registries	https://docs.dockstore.org/en/develop/advanced-topics/docker-registries.html#
Best Practices for Secure and FAIR Workflows	https://docs.dockstore.org/en/develop/advanced-topics/best-practices/best-practices-secure-fair-workflows.html
Checker Workflows	https://docs.dockstore.org/en/develop/advanced-topics/checker-workflows.html
Creating Snapshots and Requesting DOIs	https://docs.dockstore.org/en/develop/advanced-topics/snapshot-and-doi.htm
Dockstore GitHub Apps documentation	https://docs.dockstore.org/en/develop/getting-started/github-apps/github-apps.html
BCC2020 Workshop: Introduction to Docker and Descriptors	https://github.com/dockstore/bcc2020-training

Links to example content available on Dockstore and mentioned in text:

BD Catalyst Organization	https://dockstore.org/organizations/bdcatalyst
AnVIL Organization	https://dockstore.org/organizations/anvil
Broad Institute Organization	https://dockstore.org/organizations/BroadInstitute
nf-core Organization	https://dockstore.org/organizations/nfcore
PCAWG Organization	https://dockstore.org/organizations/PCAWG
Example of DOI exported from Dockstore to Zenodo	https://doi.org/10.5281/zenodo.4465061

A number of software packages, “Launch with” partners, and communities are mentioned in the main text but often lack publications and/or have substantial online documentation. Here we gather links to a number of these:

Agora	https://github.com/broadinstitute/agora
GA4GH TRS	https://www.ga4gh.org/news/tool-registry-service-api-enabling-an-interoperable-library-of-genomics-analysis-tools/
GA4GH WES	https://www.ga4gh.org/news/ga4gh-wes-api-enables-portable-genomic-analysis/
Marked (Markdown implementation)	https://marked.js.org/
DNAstack	https://www.dnastack.com/
DNAnexus	https://www.dnanexus.com/
Terra	https://terra.bio/
FireCloud	https://datacommons.cancer.gov/analytical-resource/broad-firecloud
Cancer Genomics Cloud	https://www.cancergenomicscloud.org/
AnVIL	https://anvilproject.org/
NHLBI Biodata Catalyst	https://biodatacatalyst.nhlbi.nih.gov/
CAVATICA	https://cavatica.squarespace.com/
Galaxy Project	https://galaxyproject.org/
NHGRI	https://www.genome.gov/about-nhgri/NHGRI-Vision-and-Mission
nf-core	https://nf-co.re/
Angular	https://github.com/angular and https://books.ninja-squad.com/angular
